# Structural considerations for functional anti-EGFR × anti-CD3 bispecific diabodies in light of domain order and binding affinity

**DOI:** 10.18632/oncotarget.24490

**Published:** 2018-02-14

**Authors:** Ryutaro Asano, Keisuke Nagai, Koki Makabe, Kento Takahashi, Takashi Kumagai, Hiroko Kawaguchi, Hiromi Ogata, Kyoko Arai, Mitsuo Umetsu, Izumi Kumagai

**Affiliations:** ^1^ Department of Biomolecular Engineering, Graduate School of Engineering, Tohoku University, Sendai 980-8579, Japan; ^2^ Present Address: Department of Biotechnology and Life Science, Graduate School of Engineering, Tokyo University of Agriculture and Technology, Tokyo 184-8588, Japan; ^3^ Graduate School of Science and Engineering, Yamagata University, Yonezawa 992-8510, Japan

**Keywords:** bispecific diabody, cancer immunotherapy, CD3, EGFR, functional structure

## Abstract

We previously reported a functional humanized bispecific diabody (bsDb) that targeted EGFR and CD3 (hEx3-Db) and enhancement of its cytotoxicity by rearranging the domain order in the V domain. Here, we further dissected the effect of domain order in bsDbs on their cross-linking ability and binding kinetics to elucidate general rules regarding the design of functional bsDbs. Using Ex3-Db as a model system, we first classified the four possible domain orders as anti-parallel (where both chimeric single-chain components are variable heavy domain (VH)–variable light domain (VL) or VL-VH order) and parallel types (both chimeric single-chain components are mixed with VH–VL and VL-VH order). Although anti-parallel Ex3-Dbs could cross-link the soluble target antigens, their cross-linking ability between soluble targets had no correlation with their growth inhibitory effects. In contrast, the binding affinity of one of the two constructs with a parallel-arrangement V domain was particularly low, and structural modeling supported this phenomenon. Similar results were observed with E2x3-Dbs, in which the V region of the anti-EGFR antibody clone in hEx3 was replaced with that of another anti-EGFR clone. Only anti-parallel types showed affinity-dependent cancer inhibitory effects in each molecule, and E2x3-LH (both components in VL-VH order) showed the most intense anti-tumor activity *in vitro* and *in vivo*. Our results showed that, in addition to rearranging the domain order of bsDbs, increasing their binding affinity may be an ideal strategy for enhancing the cytotoxicity of anti-parallel constructs and that E2x3-LH is particularly attractive as a candidate next-generation anti-cancer drug.

## INTRODUCTION

Although conventional monoclonal antibodies are now used as drugs to treat a variety of difficult-to-cure diseases including cancers, their use is limited by high production costs due to the requirement for a mammalian expression system and their poor penetration into tumor tissue [1, 2]. In addition, adverse clinical outcomes and data from animal studies have highlighted important limitations in their modes of action [3]. Therefore, efforts to improve the functions of antibodies have explored many strategies, one of which is the development of recombinant antibodies, especially bispecific antibodies (bsAbs). These reagents are characterized by their ability to simultaneously bind two targets; this bispecificity effectively redirects diverse effectors, including immune cells such as cytotoxic T cells, toward cancer cells. So far, only two bsAbs worldwide have been approved for clinical use, and both are designed to recruit T cells against tumor cells [4].

The difficulty of producing large amounts of homogenous bsAbs through traditional (albeit available) techniques, such as hybrid hybridomas and chemical cross-linking, has hindered their wider adoption and development as therapeutic reagents [5]; however, advances in recombinant technology have facilitated the production of homogeneous bsAbs. Numerous recombinant formats have been designed and reported, ranging from whole IgG-like molecules to small recombinant reagents, such as diabodies (Dbs) [6], single-chain diabodies [7], tandem single-chain variable fragments [8], and various other derivatives [9]. Among the large-format constructs designed, bispecific tetravalent molecules are produced by using Fc-mediated dimerization and carry two binding sites for each antigen, which imparts increased avidity. A frequent approach for producing a tetravalent bsAb is to substitute the Fab arm in IgG with a bispecific single-chain diabody or tandem single-chain variable fragment [9].

Several previous reports have suggested that the order or stability of the V domains is important to increase the functionality and applicability of bsAbs regardless of whether they are small recombinant reagents or IgG-like tetravalent ones [10, 11]. For example, bispecific Dbs (bsDbs) have four possible domain orders, and Lu et al. showed that the order of the V domain of bsDbs affects their antigen-binding activity [12]. In this regard, we previously described the construction of a functional humanized bsDb that targets epidermal growth factor receptor (EGFR) and CD3 (hEx3-Db) [13] and enhanced its cytotoxicity by rearranging the domain order of the V domain [14]. The most effective domain order of hEx3-Db was well conserved when tetravalent molecules were constructed by substituting the Fab arms in human IgG1 with hEx3-Dbs [15]. However, detailed discussions about their cross-linking ability and binding kinetics are unavailable, and general rules governing the design of functional bsDbs have yet to be developed.

**Figure 1:**
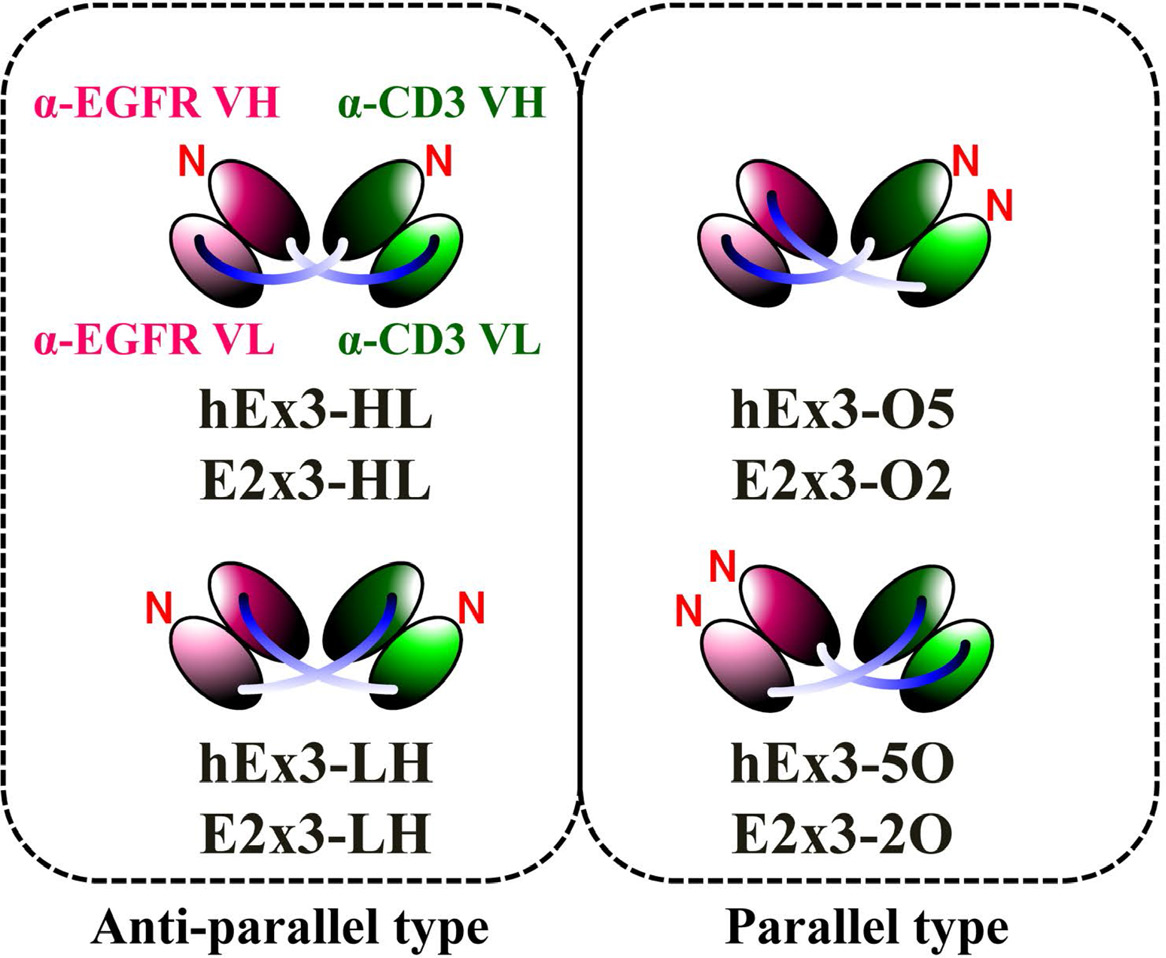
Schematic diagrams of four types of bsDbs and two types of classifications

In the present study, we first classified the four possible domain orders of bsDbs as either anti-parallel (the order of the variable heavy (VH) and variable light (VL) domains of both chimeric single-chain components is either VH-VL or VL-VH) or parallel (the domain order of the chimeric single-chain components is mixed, with one in VH-VL order and the other in VL-VH order) (Figure 1). We then prepared a series of Ex3-Db–based bsDbs representing these four domain orders. The resulting anti-parallel bsDbs cross-linked the soluble target antigens, but their crosslinking ability between soluble targets was not correlated with their growth inhibitory effects. In contrast, one of the parallel-order bsDbs showed particularly low affinities for the target antigens, and structural modeling supported this phenomenon. We obtained similar results with an E2x3-Dbs construct, in which we replaced the anti-EGFR antibody of Ex3-Dbs with another anti-EGFR clone (225). Only anti-parallel bsDbs showed affinity-dependent cancer inhibitory effects in each molecule, and E2x3-LH, in which both components are in VL-VH order, exerted the most intense anti-tumor activity *in vitro* and *in vivo*. Our results show that in addition to rearranging the domain order of bsDbs, increasing affinity may be an ideal strategy for enhancing the cytotoxicity in anti-parallel-type constructs, and that E2x3-LH is an attractive candidate for a next-generation anti-cancer drug.

## RESULTS

### Comparison of cross-linking ability by using thermodynamic analysis

We performed thermodynamic analysis to compare the abilities of hEx3-HL and -LH, two anti-parallel bsDbs, to crosslink soluble EGFR (sEGFR) and CD3εγ. Although hEx3-LH is more cytotoxic than hEx3-HL [14], the binding affinities of these two hEx3-Dbs were highly similar. Furthermore, the *K*_A_ values of both hEx3-Dbs for each target antigen remained nearly unchanged when hEx3 precomplexed with either of the target antigens was used as a ligand solution (Table 1). These results indicate that both anti-parallel domain arrangements can cross-link the soluble target antigens without steric hindrance or structural change, and the binding affinities and the cross-linking ability between soluble antigens of hEx3-HL and -LH were independent of differences in their growth inhibitory effects.

**Table 1:** *K*_A_ values obtained by using isothermal titration calorimetry

	*K*_A_ (× 10^7^ M^–1^)
hEx3-HL + EGFR	6.2 ± 0.67
hEx3-HL–CD3 + EGFR	5.1 ± 0.36
hEx3-HL + CD3	1.7 ± 0.22
hEx3-HL–EGFR + CD3	1.3 ± 0.15
hEx3-LH + EGFR	0.9 ± 0.19
hEx3-LH–CD3 + EGFR	1.2 ± 0.20
hEx3-LH + CD3	1.0 ± 0.12
hEx3-LH–EGFR + CD3	1.1 ± 0.12

### Comparison of binding constants by using SPR spectroscopy and thermodynamic analysis

**Figure 2:**
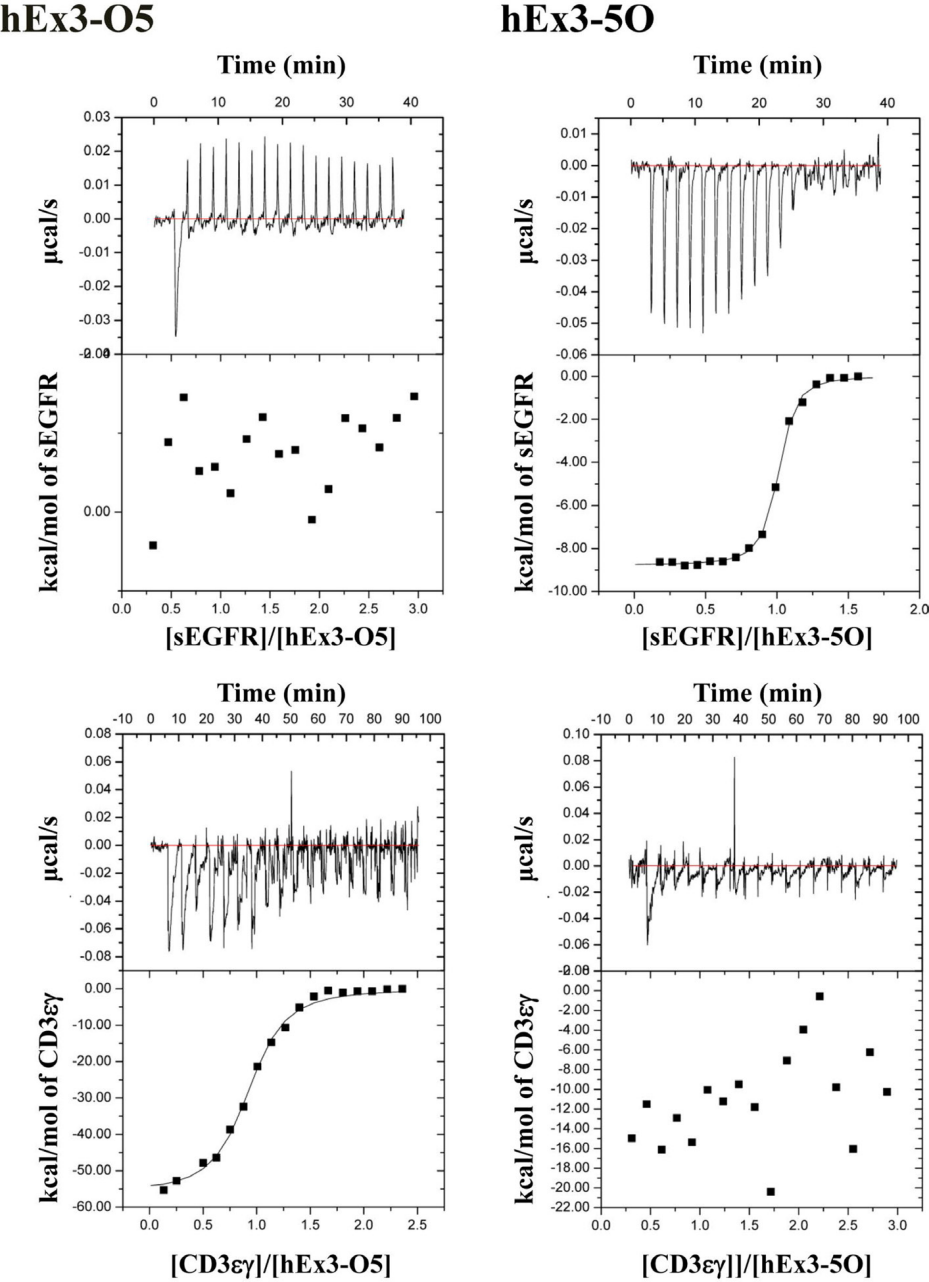
Isothermal titration calorimetry of the interactions of various bispecific diabodies with sEGFR and CD3εγ Calorimetric titration of hEx3-O5 and -5O at pH 7.2 and 25°C for sEGFR and CD3εγ is shown as representative graphs.

To further evaluate the binding affinities of hEx3-Dbs with different domain orders, including the parallel-order bsDbs hEx3-O5 and hEx3-5O, we performed kinetic analyses for immobilized sEGFR by surface plasmon resonance (SPR) imaging. The final yields of hEx3-HL, -LH, -O2, and 2O were 3.2, 0.7, 2.1, and 2.2 mg/L culture, respectively. Among the hEx3-Dbs tested, only hEx3-O5 showed low affinity to sEGFR (Table 2). Because CD3 was inactivated by immobilization on a sensor chip [16], we used isothermal titration calorimetry to perform thermodynamic analyses of CD3εγ and sEGFR. As shown in the graphs of calorimetric titration (Figure 2), under this condition, thermodynamic interactions were not observed with hEx3-O5 for sEGFR or with hEx3-5O for CD3εγ, respectively (Table 2). These results suggest that V regions located at the C-termini of bsDbs in which the domains are arranged in parallel have particularly low binding affinities.

**Table 2:** Binding parameters of bsDbs with different domain orders

	sEGFR				CD3εγ		sEGFR
	*k*_on_^a^ (× 10^4^ M^–1^s^–1^)	*k*_off_^a^ (× 10^–3^ s^–1^)	*K*_A_^a^ (× 10^7^ M^–1^)	*K*_A_^b^ (× 10^7^ M^–1^)	*K*_A_^b^ (× 10^7^ M^–1^)		*K*_A_^a^ (× 10^7^ M^–1^)
hEx3-HL	9.3	2.0	4.6 ± 0.03	6.2 ± 0.12	1.7 ± 0.22	E2x3-HL	52.7 ± 1.48
hEx3-LH	7.3	3.6	2.0 ± 0.01	0.9 ± 0.19	1.0 ± 0.12	E2x3-LH	19.0 ± 0.33
hEx3-O5	2.4	3.7	0.6 ± 0.04	n.d.	0.6 ± 0.08	E2x3-O2	0.03 ± 14.92
hEx3-5O	15.2	3.4	4.5 ± 0.01	6.5 ± 0.66	n.d.	E2x3-2O	9.9 ± 0.13

^a^Value evaluated by surface plasmon resonance spectroscopy.

^b^Value evaluated by isothermal titration calorimetry.

n.d., not determined.

To verify whether this phenomenon might be retained in other bsDbs, we prepared a series of E2x3s bsDbs with different domain orders by replacing the V regions of anti-EGFR 528 in the Ex3-Dbs with another anti-EGFR antibody, clone 225, and used SPR imaging to perform kinetic analyses of the resulting E2x3 constructs on immobilized sEGFR. The final yields of hE2x3-HL, -LH, -O2, and 2O were 0.1, 0.05, 0.03, and 0.06 mg/L culture, respectively. Binding constants of E2x3s for CD3εγ were not determined because we could not prepare enough quantity of E2x3s for ITC. Similar to the results with hEx3-Dbs, E2x3-O2, in which the V regions of anti-EGFR 225 are located at the C-terminus, had particularly low affinity against sEGFR (Table 2). These findings confirm that the C-terminal V regions in parallel-type bsDbs show low binding affinity independent of the antibody clone. Of note, the other orientations of E2x3 bsDbs had higher affinity than the hEx3-Db with the corresponding domain arrangement.

### Structural modeling

**Figure 3:**
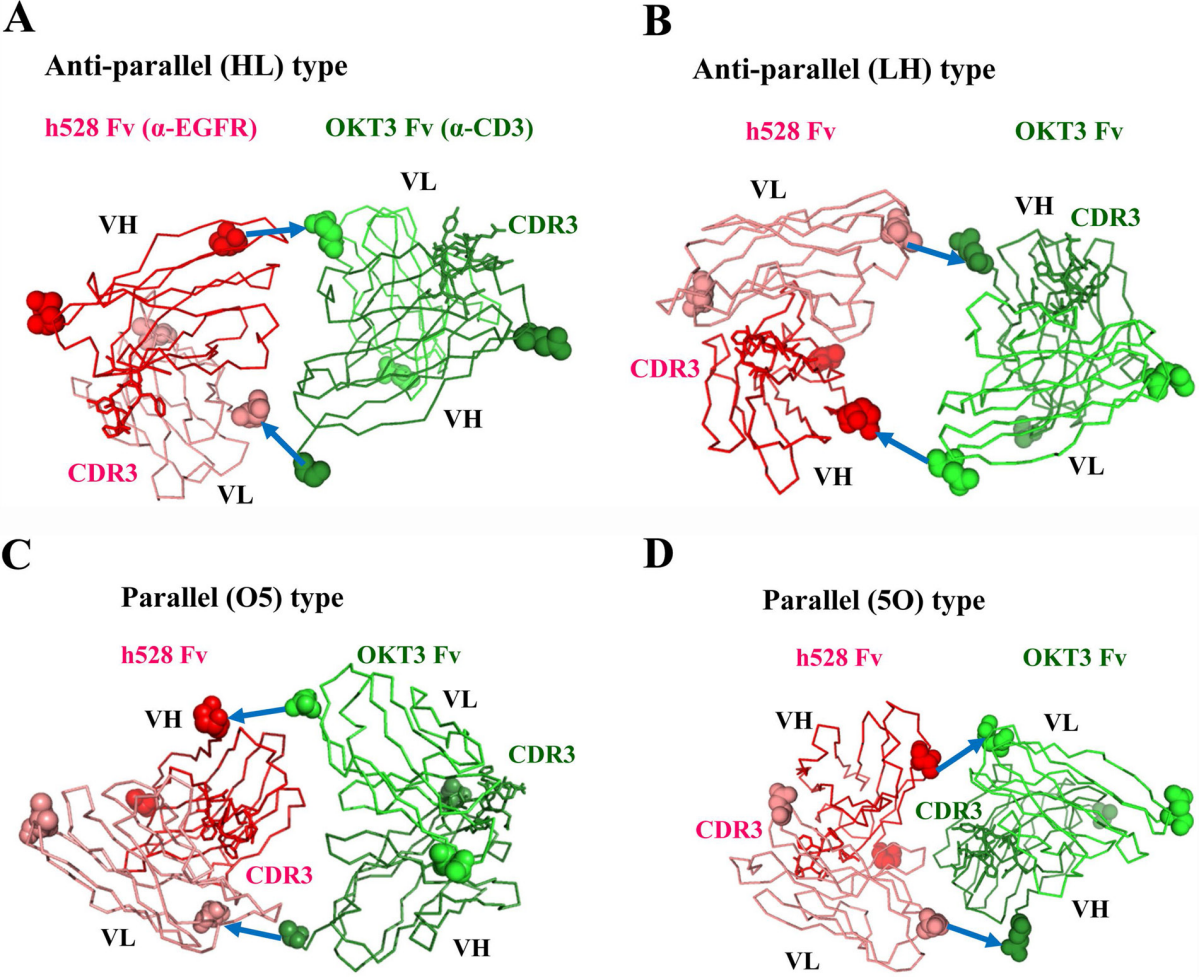
Structural models of diabody variants Anti-EGFR h528 VH and VL are shown in red and pink, respectively. Anti-CD3εγ OKT3 VH and VL are shown in green and light green, respectively. N- and C- terminal residues are shown as a CPK model. CDR-H3 loops are shown as a stick model. The GGGGS linkers are shown as arrows.

To investigate the reason why only one of the parallel-type V regions showed low affinity, we used a molecular graphics tool to construct models of the structures of the various bsDbs. The differences between the structures due to the VH compared with VL domain order in bsDbs were striking. As expected, the anti-parallel models of hEx3-HL and hEx3-LH showed that the two paratopes (CDR-H3s) of each variable fragment (Fv) face outward (Figure 3A, 3B), consistent with previously reported diabody structures [17, 18]. This orientation brings two different antigens in close proximity, resulting in a functional bridge between cancer cells and immune cells. In contrast, the structure models of the parallel-order bsDbs hEx3-O5 and hEx3-5O revealed very different orientations (Figure 3C, 3D). On the N-terminal side of Fv, anti-CD3 in hEx3-O5 and anti-EGFR in hEx3-5O partially overlaps the paratope surface on the C-terminal side of Fv. Thus, consistent with the results from the affinity assay, antigen binding by the C-terminal V regions in parallel-arrangement bsDbs (e.g., anti-EGFR Fv in hEx3-O5) is impeded due to steric hindrance from the N-terminal side of Fv.

### Effect of the domain order of E2x3 on growth inhibition and comparison with hEx3s

**Figure 4:**
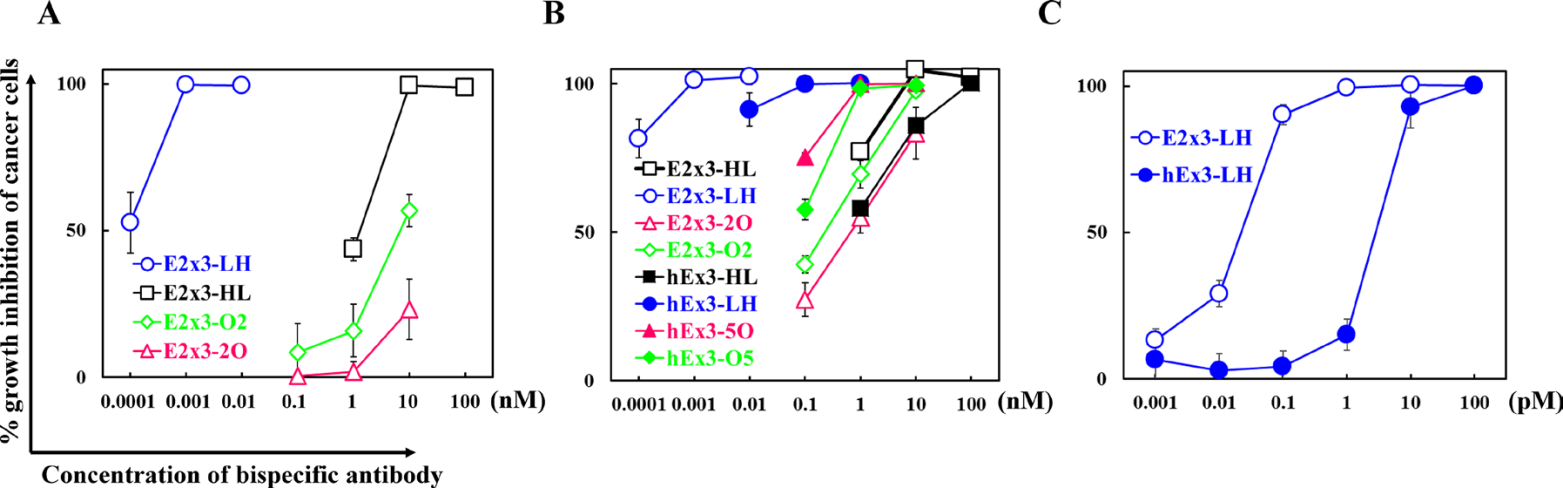
Growth inhibition of EGFR-positive TFK-1 cells by each bispecific diabody (bsDb) The bsDb hEx3s and T-LAK cells were added to TFK-1 cells (T-LAK:TFK-1 ratio, 5:1). Comparison (**A**) among E2x3-Dbs, (**B**) among E2x3-Dbs and hEx3-Dbs, and (**C**) between E2x3-LH and hEx3-LH. Data are presented as mean ± 1 SD and are representative of at least three independent experiments.

To evaluate the influence of the domain order on the inhibition of human carcinoma cell growth, we analyzed the four fractionated E2x3 dimers in cell proliferation colorimetric assays. Consistent with previous data [14], the LH type showed the highest growth inhibitory effects (Figure 4A). Comparison among all eight E2x3-Dbs and hEx3-Dbs further supported the superiorities of the LH domain order in regard to cytotoxicity (Figure 4B). In addition, the greater dose-dependent cytotoxicity of E2x3-LH compared with hEx3-LH was confirmed under conditions of decreased sample concentration (Figure 4C). Although the HL-type E2x3 bsDb was more cytotoxic than the hEx3-Db with the same domain arrangement, the E2x3-O2 and -2O constructs were less growth inhibitory than hEx3-O5 and -5O, respectively. These results suggest that increasing the affinity of anti-parallel bsDbs might enhance their individual cancer inhibitory effects. However, because the cytotoxicity of parallel-arrangement bsDbs is independent of their affinity, estimating their functionality in advance of *in vivo* testing will be difficult.

### Effect of E2x3 domain order on cytokine production

**Figure 5:**
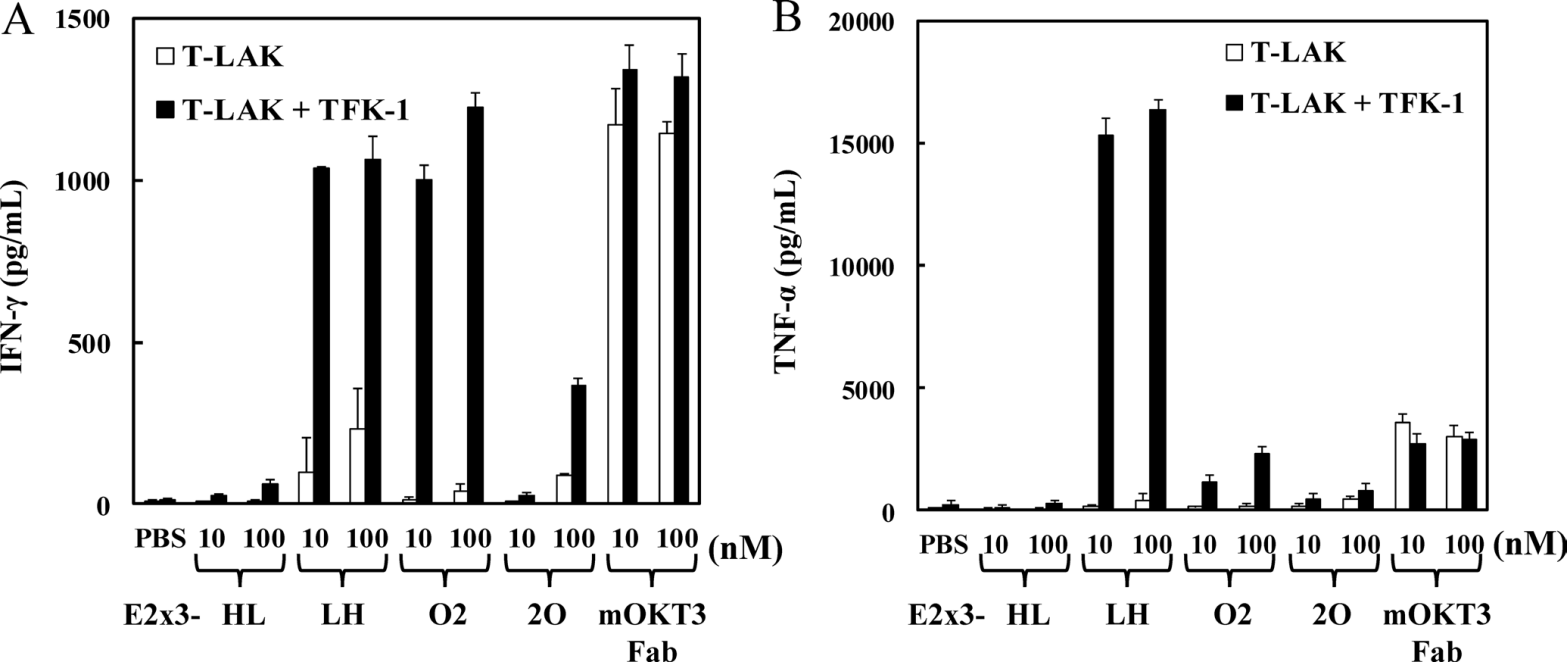
E2x3-Dbs–mediated cytokine production by T-LAK cells Concentrations of (**A**) IFN-γ and (**B**) TNF-α were evaluated by using enzyme-linked immunosorbent assays.

Our previous data showed that the structural superiority of hEx3-LH was associated with increased cytokine production by effector cells, thus enhancing the cancer growth inhibitory effect of the construct [14]. To investigate whether the marked cytotoxicity of E2x3-LH was similarly correlated with cytokine production by effector cells, we analyzed the concentrations of IFN-γ and TNF-α in the supernatant of T-LAK cells cultured with either E2x3 construct in the presence or absence of TFK-1 cells. The production of IFN-γ by T-LAK cells was similar among E2x3-LH, E2x3-O5, and OKT3 Fab; however, the amount of production was relatively low (Figure 5A). In contrast, E2x3-LH stimulated high-level production of TNF-α, which might contribute to the superior growth inhibitory effect of this construct (Figure 5B). In addition, unlike OKT3 Fab, E2x3-LH induced cytokine production only in the presence of target cells; therefore E2x3-LH might be expected to cause few side effects.

### Comparison of *in vivo* antitumor effects of E2x3-LH with hEx3-LH

**Figure 6:**
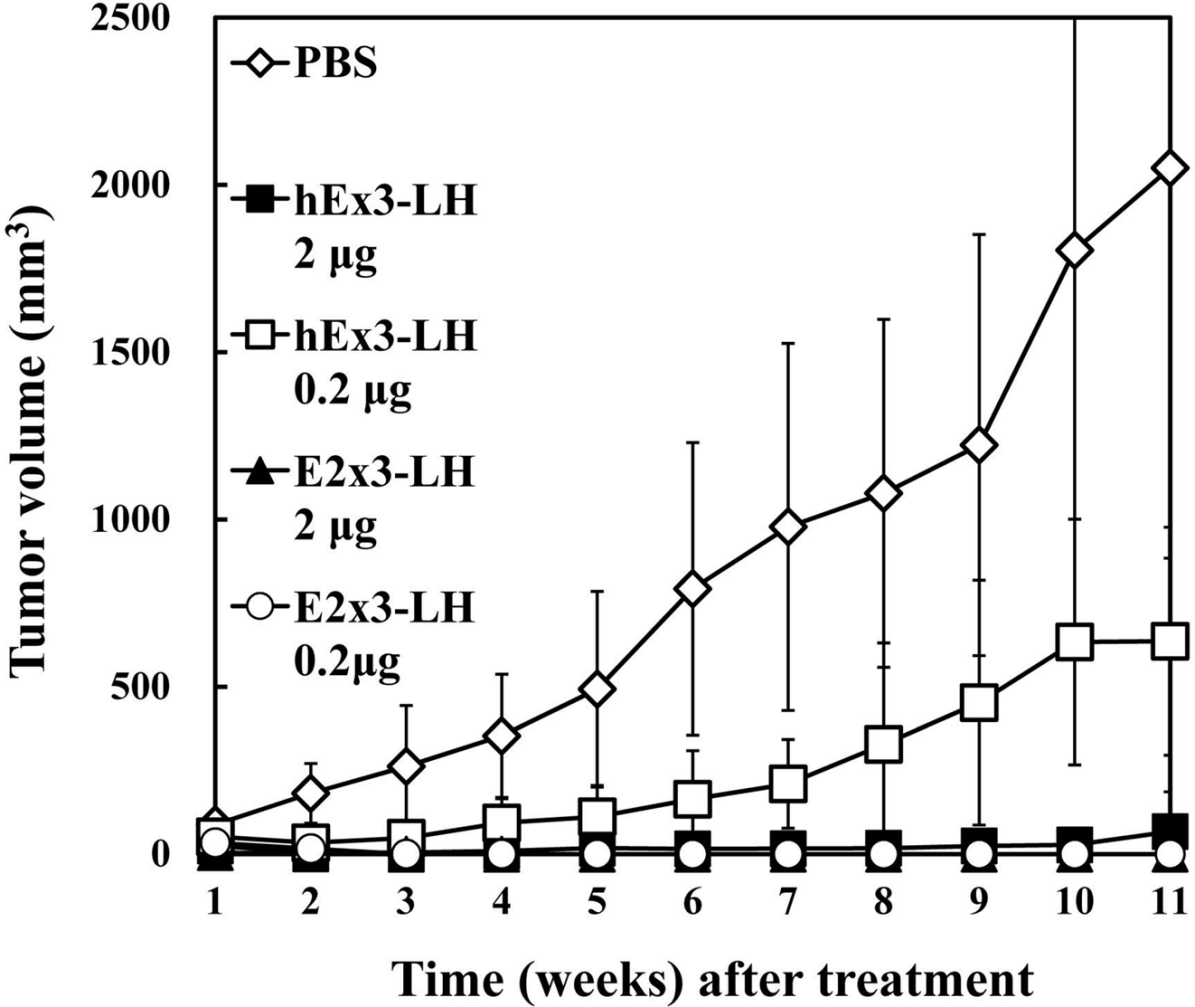
*In vivo* antitumor effects of E2x3-LH Data are given as the median tumor volume (bar, SEM) from each treatment group.

To compare *in vivo* antitumor effects of E2x3-LH and hEx3-LH, we transplanted mixtures of TFK-1 cells and T-LAK cells into SCID mice, which we then treated for four days with the bsDbs. Compared with the vehicle control, treatment with 2 μg of hEx3-LH or 2 μg of E2x3-LH markedly inhibited tumor growth in SCID mice (Figure 6). However, whereas 0.2 μg of hEx3-LH was only moderately growth inhibitory, 0.2 μg of E2x3-LH was as effective as the 2-μg dose. Therefore, in addition to rearrangement of the domain order of bsDbs, increasing their binding affinity may be an ideal strategy for enhancing the cytotoxicity of anti-parallel constructs.

## DISCUSSION

The construction of bsAbs is a practical way to develop highly functional next-generation therapeutic antibodies, especially as breakthrough anti-cancer drugs. Although there have been only two examples of approved therapeutic antibodies with fully non-natural formats, both are bsAbs. Many recombinant formats—ranging from relatively small molecules such as tandem single-chain variable fragments to large IgG-like molecules—have been proposed [9]. However, which formats are most applicable as clinical reagents remains unknown. In fact, the approved therapeutic bsAb catumaxomab is IgG-like in format, whereas the other approved compound, blinatumomab, is a tandem single-chain variable fragment construct. Moreover, although several previous reports have suggested that the order or stability of each V domain in bsAb constructs is important, a platform for producing functional and applicable bsAbs has not yet been established [10, 11].

We previously described the construction of a functional humanized bsDb that targets EGFR and CD3 (hEx3-Db) [13] and enhanced its cytotoxicity by rearranging the domain order of the V domain, and also confirmed that cancer inhibitory effects against several cell lines including with low EGFR expression and in the case where unstimulated lymphocytes were applied as effector cells [14]. We also reported the cytotoxic enhancement of Fc fusion format of hEx3 by rearranging the order of the V domain [15]. However, detailed discussion about their cross-linking ability, binding kinetics, and the general rule on design of functional bsDbs have not yet occurred. Here, we first classified bsDbs as anti-parallel and parallel types (Figure 1). Our previous data showed that, among the four possible domain orders for hEx3-Dbs, the LH-type had the highest cancer growth inhibition activity and HL-type the lowest, even though both are anti-parallel constructs. Thermodynamic analysis revealed that the LH-type and HL-type are similar in binding affinity and their ability to cross-link two soluble target antigens (Table 1). In contrast, the binding affinity of parallel-order bsDbs decreased or disappeared in the V regions located at the C-terminus of hEx3-Dbs (Table 1 and Figure 2). Consistent with these findings, another parallel-type bsDb, E2x3-O2, showed particularly low affinity against sEGFR (Table 1).

Structural modeling well supported these findings. That is, anti-parallel models (hEx3-HL and hEx3-LH) showed that the two CDR-H3s of each Fv face outward and thus are able to bind two targets, whereas parallel models (hEx3-O5 and hEx3-5O) revealed that the N-terminal Fvs cover the CDR-H3s in the C-terminal Fvs to interfere with their binding abilities (Figure 3). However, the growth inhibitory effects of bsDbs have little correlation with the binding affinity to each soluble target antigen. For example, the cancer growth inhibitory effects of anti-parallel bsDbs with specificity for EGFR and CD3 were affinity-dependent in each molecule and were highest for LH-ordered constructs (Tables 1 and 2, Figure 4). In contrast, even though the C-terminal Fvs had low affinity, all parallel-type bsDbs induced growth inhibitory effects, but the mechanism underlying this effect was unclear and the general rules could not find out (Tables 1 and 2, Figure 4). Structural dynamic changes may be caused after accessing and binding to target cells in parallel types of bsDb; however, it is difficult to estimate their functions. Our results suggest that anti-parallel types are predictable and improvable formats by domain rearrangement and/or affinity maturation in constructing functional bsDbs.

Similar to hEx3-LH in our previous study [14], E2x3-LH induced cytokine production, especially TNF-α, from T-LAK cells in the presence of target cells with higher amount than that in hEx3-LH (Figure 5). The higher affinity of E2x3-LH than hEx3-LH may contribute to this difference. Unlike that of OKT3 Fab, this target-cell–dependent cytokine production of E2x3-LH is expected to induce minimal side effects *in vivo*. Finally, compared with hEx3-LH, E2x3-LH exerted higher growth inhibitory effects in a tumor-inoculated SCID mouse model (Figure 6). Although the yield of E2x3-LH was still low for therapeutic application, we are working to optimize culturing conditions and/or production process for increasing the productivity.

The two parental anti-EGFR antibodies, 528 in hEx3 and 225 in E2x3, target similar epitope regions, compete with each other [19], and give similar binding constants, *K*_A_ = 81.7 × 10^7^ M^–1^ [20] and 43.5 × 10^7^ M^–1^ [21], respectively; however, the humanization of murine 528 caused major reduction of affinity [20], resulting in the lower affinities of hEx3-Dbs than those of E2x3-Dbs. We recently used site-directed mutagenesis to enhance the affinity of hEx3-HL and confirmed the affinity-dependency of the cytotoxicity of hEx3-HL [22]. To assess the stringency of the dependency between the affinity of anti-parallel bsDbs and their effects, we are now using the same method to increase the affinity of hEx3-LH and similar results are being obtained (manuscript in preparation). Although their features of 528 and 225 such as binding angles against target cells may differ somewhat and thus account for their different growth inhibitory effects, these results show that affinity is one of important factors for enhancing individual cancer inhibitory effects in anti-parallel bsDbs.

We report here the affinity-dependent cytotoxicity of bsDbs in which the domain order is anti-parallel, and we show the superiority of LH-type bsDbs with specificity for EGFR and CD3. The structural flexibility of the LH compared with HL arrangement has been discussed previously [18] and may contribute the high functionality of LH-type bsDbs. To confirm the versatility of these findings, we have to construct and evaluate bsDbs with other antigen specificities. Taken together, our results show that in addition to rearranging the domain order of bsDbs, increasing their binding affinity may be an ideal strategy for enhancing the cytotoxicity of anti-parallel constructs and that E2x3-LH is attractive as a candidate next-generation anti-cancer drug.

## MATERIALS AND METHODS

### Construction of expression vectors for bsDbs with different domain orientations

We previously described the construction of bacterial co-expression vectors for humanized anti-EGFR × humanized anti-CD3 bsDbs (hEx3) with different domain orders [14]: pRA-hEx3-HL for hEx3-HL, in which both chimeric single-chain components are in VH–VL order; pRA-hEx3-LH for hEx3-LH, in which both chimeric single-chain components are in VL–VH order; pRA-hEx3-O5 for hEx3-O5, in which both V regions of anti-CD3 antibody OKT3 are located at the N-terminus; and pRA-hEx3-5O for hEx3-5O, in which both V regions of anti-EGFR 528 are located at the N-terminus. The anti-EGFR antibody, clone 225, is the parental antibody of the approved therapeutic antibody cetuximab. Using the same procedure as for hEx3s and the V regions of anti-EGFR clone 225, we constructed bacterial co-expression vectors for mouse anti-EGFR × humanized anti-CD3 bsDbs (E2x3) with different domain organization: pRA-E2x3-HL for E2x3-HL, in which both chimeric single-chain components are in VH–VL order; pRA-E2x3-LH for E2x3-LH, in which both chimeric single-chain components are in VL–VH order; pRA-E2x3-O2 for E2x3-O2, in which both V regions of OKT3 are located at the N-terminus; and pRA-E2x3-2O for E2x3-2O, in which both V regions of 225 are located at the N-terminus. All HL and LH constructs were designated as anti-parallel–type bsAbs, whereas O2, 2O, O5, and 5O constructs were designated as parallel-arrangement bsAbs.

### Preparation of bsDbs

The hEx3 and E2x3 series of bsAbs, each representing the four different possibilities for domain order, were prepared by using the bacterial expression system we described previously [14]. Briefly, the constructs were expressed individually in *E. coli* strain BL21 Star (DE3) (Life Technologies, Carlsbad, CA, USA) and purified from bacterial supernatant and periplasmic fractions by using immobilized metal-affinity chromatography. Gel filtration analysis (Hiload Superdex 200-pg column 26/60, GE Healthcare Bio-Science, Piscataway, NJ, USA) was used to fractionate the dimers of each bsDb. The column was equilibrated with phosphate-buffered saline (PBS), and then purified bsDb was loaded onto the column at a flow rate of 2.0 mL/min.

### Isothermal titration calorimetry

The interactions of bsDbs with sEGFR or CD3εγ were analyzed thermodynamically by using microtitration calorimetry (ITC200 system, GE Healthcare) [23]. The methods for the expression and purification of sEGFR and CD3εγ have been described previously [20] [24]. Each sample in PBS (pH 7.2) was placed in a calorimeter cell and titrated with sEGFR or CD3εγ in the same buffer at 25 °C. The ligand solution was injected 20 times in 2-μL portions during 120 s for sEGFR and 300 s for CD3εγ. Data acquisition and subsequent nonlinear regression analyses were done according to a simple binding model, by using the ORIGIN software package (GE Healthcare). To test the ability of each bsDb to cross-link sEGFR and CD3εγ, sEGFR or CD3εγ was injected 20 times in 2-μL portions into each bsDb solution precomplexed with CD3εγ or sEGFR, respectively.

### Surface plasmon resonance spectroscopy

The interactions between sEGFR and bsDbs were analyzed by SPR spectroscopy (Biacore 2000, GE Healthcare). The methods for the expression and purification of sEGFR have been described previously [20]. sEGFR was immobilized on the cells in a CM5 sensor chip to a maximum of 2447 resonance units for hEx3s and 1318 resonance units for E2x3s. Various concentrations of bsDbs in PBS containing 0.005% Tween 20 were allowed to flow over the bound sEGFR at a flow rate of 20 μL/min at 25 °C. Both the association and dissociation times were 110 s. The surface was regenerated with 10 mM glycine–HCl (pH 2.0) with no loss of activity. The data were referenced by subtracting the response of a blocked blank cell. BIAevaluation software (GE Healthcare) was used to analyze the data. Kinetic parameters were calculated through global fitting analysis, with the assumption of a 1:1 Langmuir binding model.

### Molecular modeling

All hEx3-Dbs models for HL-, LH-, O5-, and 5O-type constructs were generated manually by using PyMOL. Although we previously analyzed the X-ray crystal structure of h528 Fv (1WT5) [20], that of humanized OKT3 Fv, a component of hEx3-Dbs, is still unknown; therefore the X-ray crystal structure of mouse OKT3 Fv (1SY6) was used for modeling [25]. The relative orientations of the two Fvs were determined so that the distance between the C-terminus of VH or VL and the N-terminus of VL or VH was less than 16 Å, which is the maximal length of the GGGGS connecting linker.

### *In vitro* growth inhibition assay

A human bile duct carcinoma (TFK-1) cell line established in our laboratory [26] was used as the target cancer cells in this study. TFK-1 cells were cultured in RPMI 1640 medium supplemented with 10% fetal bovine serum, 100 U/mL penicillin, and 100 μg/mL streptomycin. Lymphokine-activated killer cells with the T-cell phenotype (T-LAK cells) were induced as previously reported [27]. In brief, peripheral blood mononuclear cells (1 × 10^6^ cells/mL) were cultured for 48 h in medium supplemented with 100 IU/mL recombinant human IL-2 (Shionogi Pharmaceutical Co., Osaka, Japan) in a culture flask (A/S Nunc, Roskilde, Denmark) that was precoated with OKT3 IgG (10 µg/mL).

The *in vitro* inhibition of cancer cell growth was evaluated by using a 3-(4,5-dimethylthiazole-2-yl)-5-(3-carboxymethoxyphenyl)-2-(4-sulfophenyl)-2*H*-tetrazolium inner salt assay kit (CellTiter 96 AQueous Non-Radioactive Cell Proliferation Assay; Promega, Madison, WI, USA) as reported previously [27].

### Enzyme-linked immunosorbent assay

bsDbs (final concentrations, 10 and 100 nM) were co-cultured with T-LAK cells (5 × 10^4^) in the presence or absence of overnight-adhered TFK-1 cells (5 × 10^3^) in 96-well plates. Supernatants were harvested and used in enzyme-linked immunosorbent assays according to the manufacturer’s instructions (ELISA Ready-SET-Go!, Bay Bioscience, Hyogo, Japan) after 20 h of co-culture for human IFN-γ or after 16 h of co-culture for human TNF-α.

### *In vivo* tumor models

For each mouse, 1.0 × 10^7^ T-LAK cells were mixed with 5 × 10^6^ TFK-1 cells in a final volume of 0.15 mL of PBS. The mixture was injected subcutaneously into the dorsal thoracic wall of 6-week-old female severe combined immunodeficient (SCID) mice (CLEA Japan, Tokyo, Japan). Starting at 1 h after TFK-1 inoculation, mice (*n* = 5 per group) were injected intravenously with bsDb or PBS at the indicated doses; this treatment was repeated once daily for 3 consecutive days. Tumor size was measured weekly by using calipers, and the approximate tumor volume (V, in mm^3^) was calculated from linear measurements of the width (A, in mm) and length (B, in mm) as follows: V = (A^2^ × B)/2. Experiments involving mice were reviewed by the Committee on Ethics in Animal Experiments of Tohoku University and were performed under the Guidelines for Animal Experiments of Tohoku University and according to the laws and notifications of the Japanese government.

## Abbreviations

bsAb: bispecific antibody
bsDb: bispecific diabody
EGFR: epidermal growth factor receptor
Fv: variable fragment
VH: variable heavy domain
VL: variable light domain
PBS: phosphate-buffered saline
sEGFR: soluble EGFR
SPR: surface plasmon resonance
T-LAK cells: lymphokine-activated killer cells with the T-cell phenotype
